# A Comparison between the Implant Stability Quotient and the Fractal Dimension of Alveolar Bone at the Implant Site

**DOI:** 10.1155/2018/4357627

**Published:** 2018-10-15

**Authors:** Tomasz Kulczyk, Agata Czajka-Jakubowska, Agnieszka Przystańska

**Affiliations:** ^1^Section of Dental Radiology, Department of Biomaterials and Experimental Dentistry, Poznań University of Medical Sciences, Poznań, Poland; ^2^Department of Oral Rehabilitation, Division of Prosthodontics, Poznań University of Medical Sciences, Poznań, Poland

## Abstract

**Objectives:**

Fractal analysis of the radiographic pattern of bone has been used to evaluate its quantitative properties. However, the relation between initial implant stability and quality of bone remains unclear. The objective of this study was to evaluate RFA values in relation to the fractal dimension of bone where the implant was inserted.

**Material and Methods:**

A total of 50 two-stage dental implants were placed in the maxilla and mandible of 32 patients. After implant placement, an implant stability quotient (ISQ) was measured in two perpendicular planes. On intraoral digital periapical radiographs, three 35x35 pixels' regions of interest (ROIs) were chosen covering the bone adjacent to the neck (ROI 1), middle (ROI 2), and apical (ROI 3) part of the implant, respectively. For every ROI, a fractal dimension (FD) was calculated. A linear correlation, as well as a logistic regression analysis, was used to identify a possible relation between the ISQ and FD values for every ROI in the maxilla and mandible.

**Results:**

The ISQ and FD values were found to be correlated at ROI 1 for the maxilla. There was no linear correlation between ISQ and FD values in any of the three ROIs in the mandible. However, logistic regression analysis showed that in ROI 1 and ROI 3 the values of FD and ISQ are statistically important and may be used to express the difference between maxilla and mandible.

**Conclusion:**

The fractal dimension of alveolar bone measured from intraoral digital radiographs alone may be an insufficient parameter to determine initial implant stability.

## 1. Introduction

The ability to evaluate osseointegration is a valuable diagnostic and clinical tool in implant dentistry [1]. The resonance frequency analysis (RFA) provides valuable clinical objective data of implant stability [2, 3]. Resonance frequency analysis (RFA) is a method used to determine stability of dental implants in the alveolar bone. In RFA technique a probe is generating and sending magnetic pulses towards a small metal rod temporarily attached to the implant. The degree of rod vibration is recorded by the probe as its resonance frequency and expressed as implant stability quotient (ISQ) value. The RFA also detects a substantial increase or decrease in the stability of the implant, giving a clear ability to measure implant-bone contact and makes clinical comparisons during clinical follow-up [4]. However, it was reported [5] that RFA used as a single method is not suitable for the evaluation of implant stability.

Primary implant stability is the condition sine qua non for successful long-term treatment outcomes for osseointegrated implants [6, 7].

Successful implant procedure depends on the ability of the clinician to assess the degree of primary implant stability and its changes along with bone remodelling [2]. The possibility of implant stability prediction based on quantitative measurements of bone density at planned implant sites with the use of diagnostic software has been investigated [8–10]. It is emphasized that finding a method, which enables prediction of primary impact stability in the alveolar bone and finally helps in determining the healing period and increases the success rate, would be beneficial [11, 12].

Stability of the implant depends on the quality and quantity of bone, the surgical technique, and implant characteristics [13–16]. Poor bone quality and quantity have a major impact on the long-term failure rate of implants; however, the relationship between initial implant stability and quality of bone remains unclear [17]. Marquezan et al. [18] believe that the evidence to support the relationship between bone density and implant primary stability is weak and needs to be improved to produce stronger evidence. It should be emphasized that bone quality is broadly defined and includes bone density, that is, only one factor of bone quality [7]. Bone density is, in fact, the most often addressed if the quality of bone is evaluated.

Computerized tomography is commonly used for evaluation of the bone quality in clinical practice [19]. It has been reported that bone density values obtained from CBCT give predictable data about implant stability [20]. Nonetheless, a correlation between bone density measurements around implant sites in 3D reconstructions of CBCT and implant stability assessed in resonance frequency analysis (RFA) is disputable [11, 21, 22].

We hypothesize that implant stability measured by means of RFA can be possibly predicted based on quantitative measurements of bone density at planned implants sites. Thus the objective of the study was to evaluate if RFA values are correlated to quantitative properties of bone in the peri-implant region obtained by means of fractal analysis of the radiographic bone pattern along the implant bed.

## 2. Material and Methods

A total of 50 two-stage titanium dental implants (Bredent Bluesky, Germany) were placed at the crestal level in the upper and lower jaws of 32 healthy, nonsmoking patients using an open, full thickness flap protocol. Implants were placed by one operator in lateral regions of nonaugmented bone. Directly after implant placement, RFA measurements of implant stability were performed, in two perpendicular transverse and longitudinal planes, using Osttell ISQ (OsstellAB, Sweden) unit which expresses the stability of implant as implant stability quotient (ISQ). Following the placement of implants an intraoral digital periapical radiograph was taken, using PSP plates (Digora Optime) and a right angle technique film holder (Rinn Dentsply). The radiographs were then exported in BMP format for further processing and analysis utilizing the ImageJ program (https://imagej.nih.gov/ij). Three 35x35 pixels' regions of interest (ROI) were created on every radiograph, covering the bone adjacent to the neck (ROI 1), middle (ROI 2), and apical (ROI 3) part implant, respectively. The top of ROI 1 was situated 1.5 mm from the top of the implant, the middle part of ROI 2 in the middle of the total length of the implant, and the bottom of ROI 3 at the line marking the end of the implant. When ROIs were created, great care was taken not to include the implant itself.

**Figure 1:**
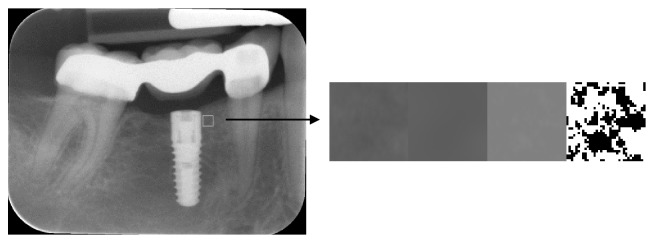
An intraoral image with marked 35x35 pixels' ROI in the neck region of implant and steps of ROI image conversion for fractal dimension analysis. Images from left to right: ROI extracted from image; blurred image with 5-pizel Gaussian filter; subtracted and normalized image; conversion into binary image.

The ROIs obtained by this method had been cropped from the original X-ray image and were converted into binary images for fractal analysis in a modified way as described by White and Rudolph [23]. For that purpose, a 5-pixel Gaussian filter was applied to every ROI to create a blurred version of that image. The resulting image was then subtracted from the image of the original ROI. Then the image was normalized by setting the intensity mean to 128, which is the centre of the intensity range for an 8-bit image. Finally, the density-normalized image was converted into binary format. Subsequent steps of ROI extraction and conversion into binary image are presented in Figure 1. For every final binary representation of an ROI, a fractal dimension (FD) was calculated using an ImageJ Fraclac plug-in. The relationships between the FD, in ROIs 1, 2, 3, and the ISQ values for both maxilla and mandible have been analyzed and compared, to look for any possible correlations between the given values in maxillae and mandibles.

### 2.1. Statistics

Statistica software (SPSS 13.0) was used for statistical analysis. The relationships between FD, in ROIs 1, 2, 3, and ISQ values have been analyzed for the entire set of data and also separately for maxilla and mandible. A linear correlation, as well as a logistic regression analysis, was used to identify a possible relation between the ISQ and FD values.

## 3. Results

### 3.1. Fractal Dimensions of the Bone

Summarized results of fractal dimension and ISQ values for the mandible and maxilla are presented in Tables 1 and 2. For all ROIs in the maxilla, the mean value of the fractal dimension was approximately 1.61 with similar values of the standard deviation of 0.032. The mean fractal dimension for each ROI in the mandible was approximately 1.57 with a standard deviation of 0.032. The mean ISQ value in both transverse and longitudinal plane in the mandible was higher than the one for maxilla (78.36 and 79.21 versus 71.38 and 72.63, respectively). The observed differences in both FD and ISQ value were statistically significant (p values 0.007 and 0.009, respectively).

### 3.2. Fractal Dimension and ISQ Value versus Implant Position

**Figure 2:**
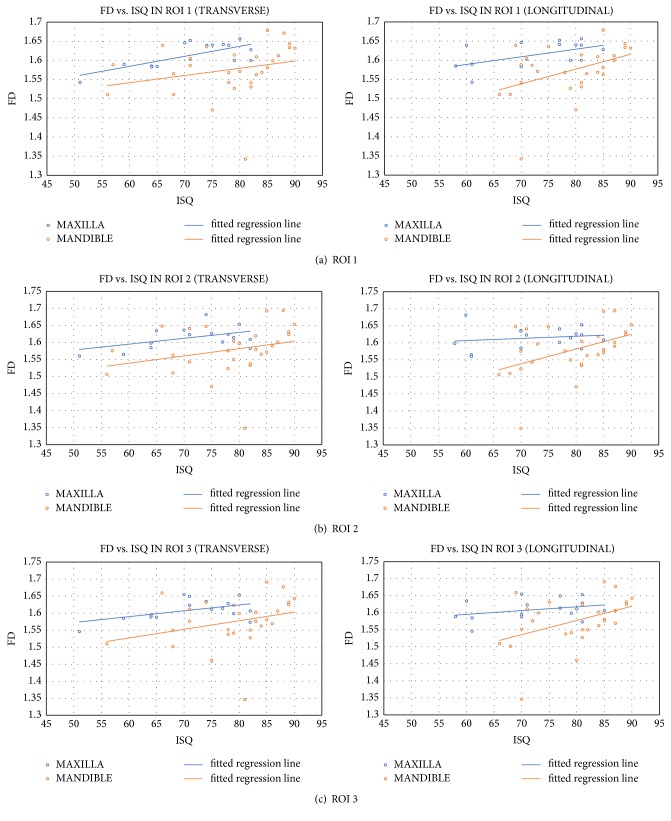
Relationship between FD and ISQ transverse (left column) and longitudinal (right column) values in ROIs 1 (a), 2 (b), and 3 (c) with fitted regression line for maxilla and mandible.

The relationship between the fractal dimension of bone in each ROI in both maxilla and mandible and the transverse or longitudinal ISQ values of initial stability of the dental implant are presented in Figure 2. A linear and positive relationship in all ROIs has been observed. The relationship is stronger for implants in the mandible. Pearson's linear correlation coefficients were performed to measure the strength of any correlation between traits and the results of such analyses are presented in Table 3.

A significant correlation was observed only for the fractal dimension in the ROI 1 of the maxilla. The strength of the correlation was considerable and in a positive direction. In other regions, the correlation was not significantly different from zero (significance level *α* =0.01).

### 3.3. Fractal Dimension as a Predictor of ISQ Value of Dental Implant Initial Stability

To test the hypothesis about the implication of the fractal dimension of bone in ROI on the ISQ transverse and longitudinal values, two models of the regression function have been used. In the first model, the dependent variable was the ISQ transverse value, while the independent variables were fractal dimension values in each of the ROIs. In the second model as the dependent variable, an ISQ longitudinal value was used, while the independent variables remained the same. In Table 4, results of statistical analysis of estimated goodness of fit are presented. F-statistic was used to test the hypothesis that none of the regression coefficient factors significantly differs from zero. In each case, F is higher than the accepted value of significance *α* = 0.01. This means that none of the models in no way explains the variability of the response of the variable. The backward elimination of variables method used for the analysis showed that no variable significantly describes the variability of any measure of stability.

## 4. Discussion

To date, many methods have been established in order to investigate the quality of the alveolar bone. Fractal analysis is a method of quantitatively measuring complex geometric structures that exhibit patterns throughout the image [24]. It is regarded as a noninvasive indicator of bone remodelling, bone regeneration, and bone loss [25, 26].

The FA of the bone tissue has been introduced as an accurate, economical, and easily available method for assessing bone trabecular patterns around the implants in different clinical situations [4, 27–29].

The usefulness of fractal dimension analysis based on the computed tomography for peri-implant bone quality evaluation has been investigated [9, 12, 30]. Although there is no consensus on the relationship between fractal dimension and trabecular bone complexity [31], it has been demonstrated that fractal analysis of the bone is associated with changes in bone density [32, 33] and reflects the partial demineralization of bone [24]. Moreover, the latter showed that fractal analysis is suitable for discrimination between anatomical location and the degree of demineralization.

In our study, fractal dimensions calculated for regions of interest in maxilla were higher than the ones calculated for regions of interest in the mandible. These observations may come from composition and pattern of cortical and cancellous bone in the alveolar process of maxilla and mandible. Denser structure of bone in mandible seems to be less complex in pattern while the maxillary one is generally seen as rich in a trabecular structure which converts to more complex pattern with higher FD.

Heo et al. [27] used fractal analysis for evaluation of the radiographic changes to the operational sites and observed that FD decreased immediately after the operation and then increased gradually. They observed that, after 12 months (equivalent to mandibular healing and bone remodeling), the FD was similar to the preoperative values and concluded that it can be used to evaluate the bony healing process.

It has been suggested that the diminishing of fractal dimension corresponds to a reduction of bone density [34]. Furthermore, Abdulhameed et al. [4] concluded that implants with low FD values may indicate a decrease in stability.

Our results show that the initial stability of the implant measured by ISQ was higher for implants placed in the lateral region of the mandible than for implants placed in the lateral region of the maxilla. Implant stability quotient comes from the conversion of resonance frequency values which can range from 3500 up to 8500 Hz to ISQ numbers. A theoretical maximum value of ISQ is 100 (with a theoretical minimum of 0) but numbers higher than 65 are considered as predictors of sufficient initial stability of the implant. In our study majority of implants exceed ISQ number of 65 but in 5 cases in maxilla and 2 cases in the mandible, the ISQ was lower with values ranging from 51 to 64 and 56 to 58 in maxilla and mandible, respectively. Later observations, although not included in this study, revealed that out of all implants with ISQ lower than 65 two in maxilla did not integrate successfully in a period of 5 months while other 3 in the maxilla and two in mandible did.

It has been suggested that fractal analysis may be used to distinguish site-specific differences [35]. In a study of Abdulhameed et al. [4], there was a statistically significant linear correlation between the ISQ values from the RF and the FD values on both sides of the implant with the mesial side of the intervention group being higher than that on the distal side. In our study, only one region of interest (namely, ROI 1 for maxilla) FD was correlated with ISQ values while for all other cases no correlation was observed. This leads us to the conclusion that, in given clinical scenario, the fractal dimension of bone calculated in the region where implant is to be placed cannot serve as a valuable predictor of initial implant stability measured by means of resonance frequency analysis.

It has been suggested that the FD acquired from panoramic radiographs may be a useful predictor of the initial dental implants stability [9, 10, 12]. The newest reports [4] confirm that the FDA could be recommended as an adjunctive quantitative method in the prediction of the implant stability with very high sensitivity and specificity. Also, Sennerby [36] concluded that examination of bone density with use of preoperative cone beam computed tomography (CBCT) may be used as an additional feature in treatment-planning software to predict primary stability. Our study revealed that although fractal analysis may be used to determine bone quantitative properties, its application for prediction of initial implant stability (expressed in ISQ values) is questionable.

## 5. Conclusions

The fractal dimension of alveolar bone measured from intraoral digital radiographs alone may be an insufficient parameter to determine initial implant stability. The prediction of implant stability may require more complex approach including morphology of cancellous and cortical bone.

## Figures and Tables

**Table 1:** Descriptive statistics of fractal dimension values for maxilla and mandible.

	Maxilla			Mandible		
Statistics	ROI 1	ROI 2	ROI 3	ROI 1	ROI 2	ROI 3

Mean	1.61428	1.6146	1.60926	1.57589	1.57839	1.57552

Median	1.6153	1.6186	1.60885	1.5809	1.57955	1.5764

Variance	0.001	0.001	0.001	0.005	0.005	0.005

SD	0.03269	0.0323	0.03045	0.06928	0.07273	0.0714

Minimum	1.54255	1.5603	1.54585	1.3423	1.34795	1.34595

Maximum	1.656	1.6817	1.6546	1.6781	1.6941	1.6911

**Table 2:** Descriptive statistics of ISQ values for maxilla and mandible.

	transverse				Longitudinal			
location	mean	min	max	SD	mean	min	max	SD

Maxilla	71.38	51	85	9.66	72.63	58	90	8.58

Mandible	78.36	56	90	8.91	79.21	66	90	8.78

**Table 3:** Pearson correlation coefficient for FD and ISQ values and their significance for maxilla and mandible.

location	FD	STATSITICS	ISQ transversal	ISQ longitudinal
maxilla	ROI 1	Pearson correlation	**∗** **0.707**	0.537
		p-value	**∗** **0.002**	0.032
	ROI 2	Pearson correlation	0.471	0.176
		p-value	0.066	0.514
	ROI 3	Pearson correlation	0.498	0.321
		p-value	0.294	0.159

mandible	ROI 1	Pearson correlation	0.253	0.461
		p-value	0.194	0.014
	ROI 2	Pearson correlation	0.273	0.452
		p-value	0.159	0.016
	ROI 3	Pearson correlation	0.293	0.43
		p-value	0.138	0.025

**Table 4:** Statistic of fit of model 1 (ISQ transverse) and 2 (ISQ longitudinal).

	Model 1	Model 2
Statistics	Value	Value

R	0.226	0.366

*R* ^2^	0.051	0.134

*Adjusted R* ^2^	-0.022	0.067

S_e_	9.304	8.229

F	0.7	2.01

p value(F)	0.558	0.128

## Data Availability

All data used to support the findings of this study are available from the corresponding author upon request.
